# INFOGRAPHIC: 2-year efficacy, durability and safety of intravitreal faricimab with treat-and-extend dosing up to 16 weeks in diabetic macular oedema (pooled results from YOSEMITE and RHINE)

**DOI:** 10.1038/s41433-024-03249-0

**Published:** 2024-07-29

**Authors:** Naomi Wijesingha, Kara Gibson, Jeffrey R. Willis, Sobha Sivaprasad

**Affiliations:** 1 UCL Institute of Ophthalmology, London, UK; 2 Moorfields Eye Hospital NHS Foundation Trust, London, UK; 3 Roche Products Ltd., Welwyn Garden City, UK; 4 Genentech Inc., South San Francisco, CA USA

**Keywords:** Retinal diseases, Outcomes research

**Fig. 1:**
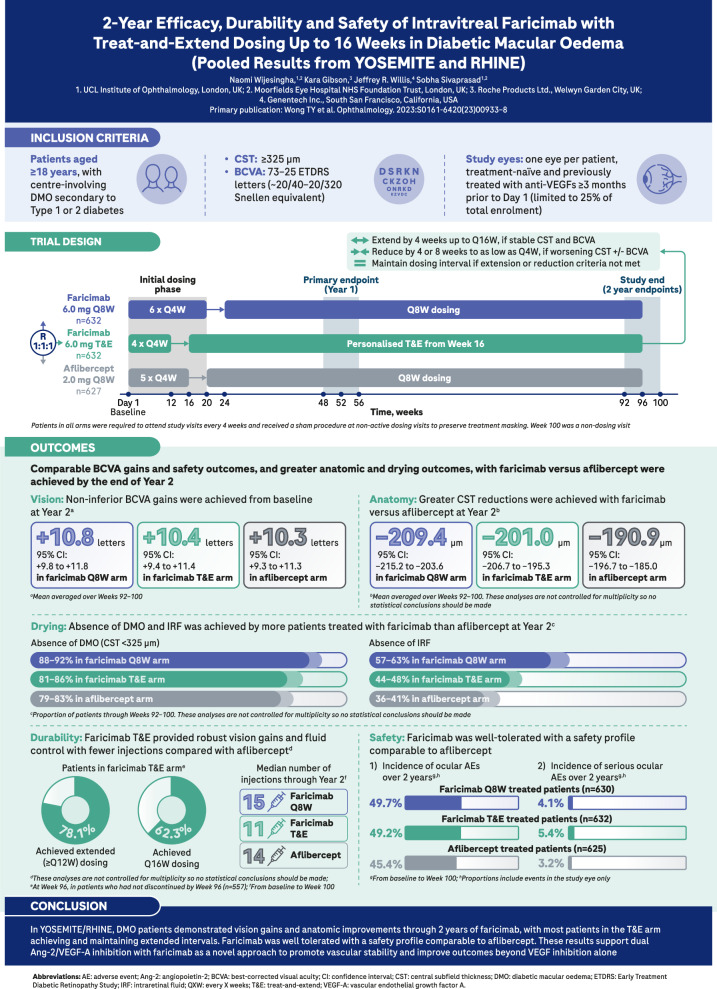
Pooled 2-year results from two Phase 3 trials (YOSEMITE and RHINE) of faricimab, a bispecific antibody targeting both Ang-2 and VEGF-A, in patients with DMO. These results support dual Ang-2/VEGF-A inhibition with faricimab as a novel approach to promote vascular stability and improve outcomes beyond VEGF inhibition alone.

Naomi Wijesingha [1,2], Kara Gibson [3], Jeffrey R. Willis [4] and Sobha Sivaprasad [1,2]✉

*Eye* (2025) 39:167–169; https://doi.org/10.1038/s41433-024-03249-0

## ACKNOWLEDGEMENTS
The authors thank Melanie Howes, Costello Medical, UK, for design support. The authors also acknowledge Ellie Fung, Costello Medical, UK, for medical writing assistance based on the authors' input and direction.

## AUTHOR CONTRIBUTIONS
All authors equally contributed to the conceptualisation and creation of the visual abstract.

## FUNDING
Funding was provided by F. Hoffmann-La Roche for the YOSEMITE/RHINE studies (NCT03622580/NCT03622593) and publication. Support for third-party assistance for this publication, provided by Ellie Fung and Melanie Howes, Costello Medical, UK, was funded by F. Hoffmann-La Roche.

## COMPETING INTERESTS
NW declares no conflicts of interest. KG is an employee of Roche Products Ltd. JRW is an employee of Genentech Inc. SS reported receiving financial support from AbbVie, Amgen, Apellis, Bayer, Biogen, Boehringer Ingelheim, Eyebiotech, Eyepoint Pharmaceuticals, Janssen Pharmaceuticals, Kriya Therapeutics, Nova Nordisk, Novartis, Ocular Therapeutics, OcuTerra, Optos, Roche, Sanofi, and Stealth Biotherapeutics, and is Editor in Chief of *Eye*.

## ADDITIONAL INFORMATION
**Correspondence** and requests for materials should be addressed to Sobha Sivaprasad.

**Reprints and permission information** is available at http://www.nature.com/reprints

**Publisher's note** Springer Nature remains neutral with regard to jurisdictional claims in published maps and institutional affiliations.

**Open Access** This article is licensed under a Creative Commons Attribution 4.0 International License, which permits use, sharing, adaptation, distribution and reproduction in any medium or format, as long as you give appropriate credit to the original author(s) and the source, provide a link to the Creative Commons licence, and indicate if changes were made. The images or other third party material in this article are included in the article's Creative Commons licence, unless indicated otherwise in a credit line to the material. If material is not included in the article's Creative Commons licence and your intended use is not permitted by statutory regulation or exceeds the permitted use, you will need to obtain permission directly from the copyright holder. To view a copy of this licence, visit http://creativecommons.org/licenses/by/4.0/.
